# Epigenetic aging is associated with aberrant neural oscillatory dynamics serving visuospatial processing in people with HIV

**DOI:** 10.18632/aging.204437

**Published:** 2022-12-15

**Authors:** Mikki Schantell, Brittany K. Taylor, Rachel K. Spooner, Pamela E. May, Jennifer O’Neill, Brenda M. Morsey, Tina Wang, Trey Ideker, Sara H. Bares, Howard S. Fox, Tony W. Wilson

**Affiliations:** 1 Institute for Human Neuroscience, Boys Town National Research Hospital, Boys Town, NE 68010, USA; 2 College of Medicine, University of Nebraska Medical Center (UNMC), Omaha, NE 68198, USA; 3 Department of Pharmacology and Neuroscience, Creighton University, Omaha, NE 68178, USA; 4 Institute of Clinical Neuroscience and Medical Psychology, Heinrich-Heine University, Düsseldorf, Germany; 5 Department of Neurological Sciences, UNMC, Omaha, NE 68198, USA; 6 Department of Internal Medicine, Division of Infectious Diseases, UNMC, Omaha, NE 68198, USA; 7 Department of Medicine, University of California San Diego, La Jolla, CA 92093, USA

**Keywords:** HIV, epigenetics, biological age, visuospatial discrimination, oscillations

## Abstract

Background: Despite effective antiretroviral therapy, cognitive impairment and other aging-related comorbidities are more prevalent in people with HIV (PWH) than in the general population. Previous research examining DNA methylation has shown PWH exhibit accelerated biological aging. However, it is unclear how accelerated biological aging may affect neural oscillatory activity in virally suppressed PWH, and more broadly how such aberrant neural activity may impact neuropsychological performance.

Methods: In the present study, participants (n = 134) between the ages of 23 – 72 years underwent a neuropsychological assessment, a blood draw to determine biological age via DNA methylation, and a visuospatial processing task during magnetoencephalography (MEG). Our analyses focused on the relationship between biological age and oscillatory theta (4-8 Hz) and alpha (10 - 16 Hz) activity among PWH (n=65) and seronegative controls (n = 69).

Results: PWH had significantly elevated biological age when controlling for chronological age relative to controls. Biological age was differentially associated with theta oscillations in the left posterior cingulate cortex (PCC) and with alpha oscillations in the right medial prefrontal cortex (mPFC) among PWH and seronegative controls. Stronger alpha oscillations in the mPFC were associated with lower CD4 nadir and lower current CD4 counts, suggesting such responses were compensatory. Participants who were on combination antiretroviral therapy for longer had weaker theta oscillations in the PCC.

Conclusions: These findings support the concept of interactions between biological aging and HIV status on the neural oscillatory dynamics serving visuospatial processing. Future work should elucidate the long-term trajectory and impact of accelerated aging on neural oscillatory dynamics in PWH.

## INTRODUCTION

With the advent of combination antiretroviral therapy, HIV is now a manageable chronic condition, and people with HIV (PWH) have a similar life expectancy to that of seronegative controls [1, 2]. However, HIV is known to cross the blood-brain barrier in the early stages of systemic viremia, which can lead to infected neural cells and increased neuroinflammation [3]. Inflammatory processes have been thought to lead to epigenetic modifications resulting in advanced aging in PWH, as well as downstream effects on cognition [4]. Many structural and functional neuroimaging studies have shown differences between PWH and controls that would be consistent with accelerated aging in PWH [5–9], although to date, studies directly linking accelerated aging in PWH to structural and functional changes in the brain have been exceedingly rare. One recent structural neuroimaging study aiming to directly link accelerated aging to HIV-related aberrations revealed common structural changes with aging, such as gray matter thinning in the posterior cortices which is accentuated in PWH who exhibit accelerated biological aging [5].

To our knowledge, no study to date has directly linked accelerated biological aging in PWH to the neuro-functional changes that occur in cognitively impaired PWH, which include deficits in visuospatial processing, attention, working memory, and motor function networks [10–19]. This paucity of work linking biological aging and functional brain activity persists despite a growing body of research showing chronological age- and disease-specific alterations to these functional networks in PWH. More specifically, impairments in visuospatial processing in PWH have been associated with decreased oscillatory theta activity and altered alpha activity recorded using magnetoencephalography (MEG) [11]. Additionally, chronological age has been shown to covary differentially with alpha and gamma neural oscillatory activity in the occipito-parietal cortices among PWH, with such alpha activity reflecting disinhibition of visual processing circuits [12]. Other studies have found differences in neural oscillatory activity related to aging in cognitively impaired PWH compared to cognitively unimpaired PWH and seronegative controls [12, 20], but none of these functional studies have included a marker of biological aging. Quantifying a biomarker of biological age could better characterize senescence, and therefore may enable more accurate representations of the aging processes occurring in the brain.

The epigenetic clock is a promising measure of biological aging in PWH, as it has been found to independently predict mortality risk [21], and it quantifies the cytosine phosphate guanine sites (CpGs) measured in human cells [22, 23]. Among PWH, advanced aging using the epigenetic clock has been identified in peripheral blood and neural tissue [24], and is linked with cognitive impairment in PWH [25]. However, it is not clear how advanced biological aging as reflected by the epigenetic clock relates to changes in the neural oscillatory dynamics serving visuospatial discrimination.

In this study, we quantified differences in the neural dynamics serving visuospatial processing among PWH and seronegative controls using advanced MEG methods and examined how these differences in neural dynamics may relate to biological age acceleration in PWH. Our measures of biological age were derived via DNA methylation analysis conducted on peripheral blood samples. Finally, we probed the link between aberrant neural dynamics and HIV disease metrics to identify phenotypes of accelerated biological aging. Our primary hypothesis was that PWH would exhibit accelerated biological aging, which would be associated with altered neural dynamics in regions serving visuospatial processing and clinical HIV indices. Specifically, we predicted that these altered neural dynamics would be in accordance with the compensation-related utilization of neural circuits hypothesis (i.e., CRUNCH) [13, 26], which suggests that older adults recruit greater neural resources relative to younger adults to perform cognitively demanding tasks.

## RESULTS

### Participant characteristics, neuropsychological, and behavioral results

**Figure 1:**
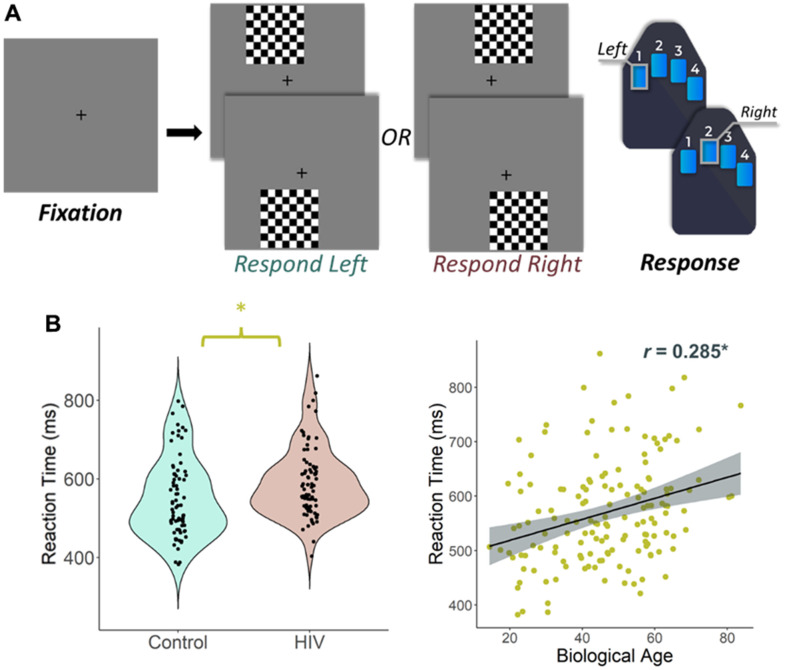
**Experimental paradigm and behavioral results.** (**A**) An illustration of the visuospatial processing task paradigm. Each trial had a fixation period lasting 2000 ms (variable ISI: 1900-2100 ms) and a stimulus-presentation period lasting 800 ms, which consisted of one of the four options displayed. Participants responded whether the checkered grid appeared to the left or to the right. (**B**) (Left) Reaction time (in ms) is displayed on the *y*-axis. There was a significant difference in reaction time by group such that people with HIV were slower to respond than controls, but they did not differ in terms of accuracy. (**B**) (Right) Biological age was associated with reaction time in all participants. *p < 0.05.

A total of 134 participants (65 PWH, 69 seronegative controls) successfully completed the visuospatial discrimination task, neuropsychological assessment, and provided a blood sample for DNA methylation analysis. The groups were similar in demographic characteristics such as chronological age (Range = 23 – 72 years), sex, race, and ethnicity, though they differed in several neuropsychological domains including memory, executive function, processing speed, and attention, and years of education achieved with the PWH performing worse than controls (Table 1). As predicted, the two groups differed in biological age when controlling for chronological age (*t*(132) = -1.79, *p* = 0.037; Table 1), which indicates greater age acceleration in PWH relative to the seronegative controls. Behavioral performance on the visuospatial processing task was similar between groups in terms of accuracy (*t*(132) = 1.35, *p* = 0.180), but seronegative controls responded faster (*M* = 543.65 ms, *SD* = 95.08) than PWH (*M* = 593.45 ms, *SD* = 93.98), *t*(132) = -3.04, *p* = 0.003 (Figure 1B, left). Greater reaction times were also significantly associated with increasing biological age, *r*(135) = 0.285, *p* = 0.001 (Figure 1B, right).

**Table 1:** Participant demographics and neuropsychological profiles.

		**HIV (*n*=65)**	**Seronegative controls (*n*=69)**	***P* value**
**Demographics**	**Chronological Age (years)**	47.48 (10.94)	44.74 (14.68)	0.221
	**Biological Age (years)**	49.27 (13.65)	44.72 (15.65)	0.037*
	**Sex (n)**	39 Male / 26 Female	34 Male / 35 Female	0.213
	**Race (n)**	42 white / 20 Black / 2 Other	49 white / 19 Black / 1 Other	0.711
	**Ethnicity (n)**	61 Not Hispanic / 3 Hispanic	65 Not Hispanic / 4 Hispanic	0.775
	**Education (years)**	14.67 (2.41)	17.10 (1.87)	<0.001
**HIV Clinical Metrics**	**CD4 Nadir (cells/μL)**	240.05 (152.79)	-	-
	**Current CD4 (cells/μL)**	828.55 (439.93)	-	-
	**Years Since HIV Diagnosis**	11.37 (6.92)	-	-
	**Years on cART**	9.35 (6.15)	-	-
**Neuropsychological Performance**	**Learning T-Score**	43.25 (10.94)	46.36 (10.77)	0.099
	**Memory T-Score**	44.99 (9.60)	49.01 (8.66)	0.012
	**Executive Function T-Score**	46.96 (7.67)	50.30 (7.45)	0.012
	**Processing Speed T-Score**	47.60 (8.13)	52.59 (6.44)	<0.001
	**Attention T-Score**	44.65 (8.74)	51.95 (8.67)	<0.001
	**Motor Dexterity T-Score**	44.32 (11.06)	47.26 (8.22)	0.089

Note. Means and standard deviations are displayed for each continuous variable. Domain T-scores were calculated by averaging the T-scores from the individual assessments comprising each cognitive domain. cART, Combination antiretroviral therapy. *One-tailed *p*-value.

### Oscillatory neural responses

**Figure 2:**
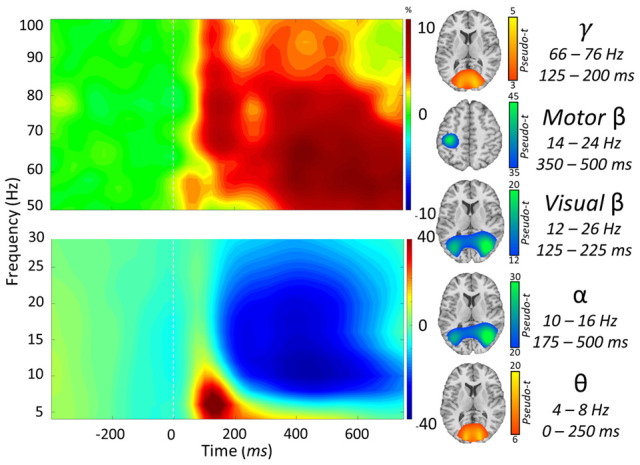
**Neural responses to the visuospatial discrimination task.** (Left): Grand-averaged time frequency spectrograms of MEG sensors exhibiting one or more significant responses. Shown from top to bottom: gamma, motor beta, visual beta, alpha, and theta activity. In each spectrogram, frequency (Hz) is shown on the *y*-axis, and time (ms) is shown on the *x*-axis. Signal power data are expressed as a percent difference from the baseline period, with color legends shown with each spectrogram. (Right): Grand-averaged beamformer images (pseudo-*t*) across all participants for each time-frequency component.

We observed robust neural oscillatory responses in five temporally and spectrally defined windows during visuospatial processing (Figure 2). These included statistically significant increases in power relative to the baseline period in the theta band (0 – 250 ms; 4 – 8 Hz), a decrease in power in the alpha band (175 – 500 ms; 10 – 16 Hz), an early decrease in power in the beta band (125 – 225 ms; 12-26 Hz), a late decrease in power in the beta band (350 – 500 ms; 14-24 Hz), and an increase in power in the gamma band (125 – 200 ms; 66 – 76 Hz). All responses were significant at *p* < 0.001 following multiple comparisons correction using nonparametric cluster-based permutation testing. At the cortex level, the theta, alpha, and early beta responses originated from the bilateral occipital regions and the late beta response originated from the left sensorimotor cortices (Figure 2, right).

### Group and biological age interactions

**Figure 3:**
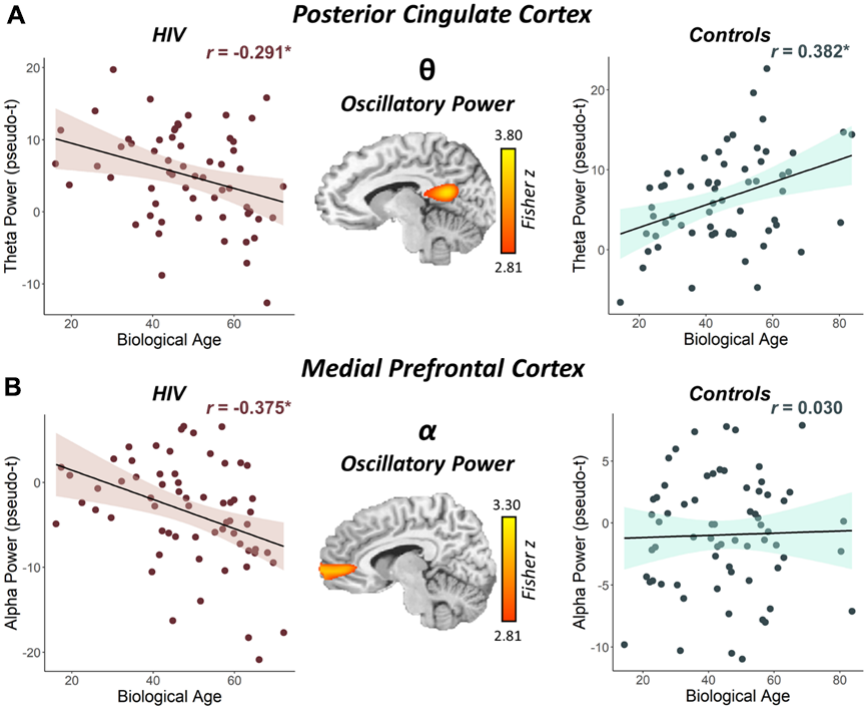
**Interaction between biological age and HIV status on the posterior cingulate theta response and the medial prefrontal cortex alpha response.** (**A**) The brain image (middle) represents the voxel-wise interaction between biological age and HIV status (i.e., people with HIV vs. seronegative controls) in oscillatory theta power in the left posterior cingulate. (**B**) The brain image (middle) represents the voxel-wise interaction between biological age and HIV status in oscillatory alpha power in the right medial prefrontal cortex. Peak voxel power values were extracted from both clusters depicted in these images and plotted as a function of biological age by HIV status to visualize the differing relationships between biological age and oscillatory neural activity. *p < 0.05.

To address our primary hypotheses, we assessed the interactive effects of biological age and HIV status on these neural responses by computing whole-brain Fisher *Z* comparisons separately for the theta, alpha, beta, and gamma band responses. In the theta band, a significant interaction between biological age and group was observed in the left posterior cingulate cortex (PCC; *z_peak_* = 3.76, *p* < 0.005, *k* = 136 voxels). Specifically, PWH had decreasing oscillatory theta power in the left PCC with increasing biological age (*r* = -0.291, *p* = 0.025), while seronegative controls had increasing theta power in the left PCC with increasing biological age (*r* = 0.382, *p* = 0.002; Figure 3A). A significant interaction between biological age and group was also found in the alpha band, and this effect was centered on the right medial prefrontal cortex (mPFC; *z_peak_* = 3.16, *p* < 0.005, *k* = 204 voxels), with PWH exhibiting decreasing oscillatory alpha power with increasing biological age (*r* = -0.375, *p* = 0.003) and controls showing no significant relationship (*r* = 0.030, *p* = 0.812; Figure 3B). There were no significant group-by-biological age interactions observed in the beta and gamma bands.

**Figure 4:**
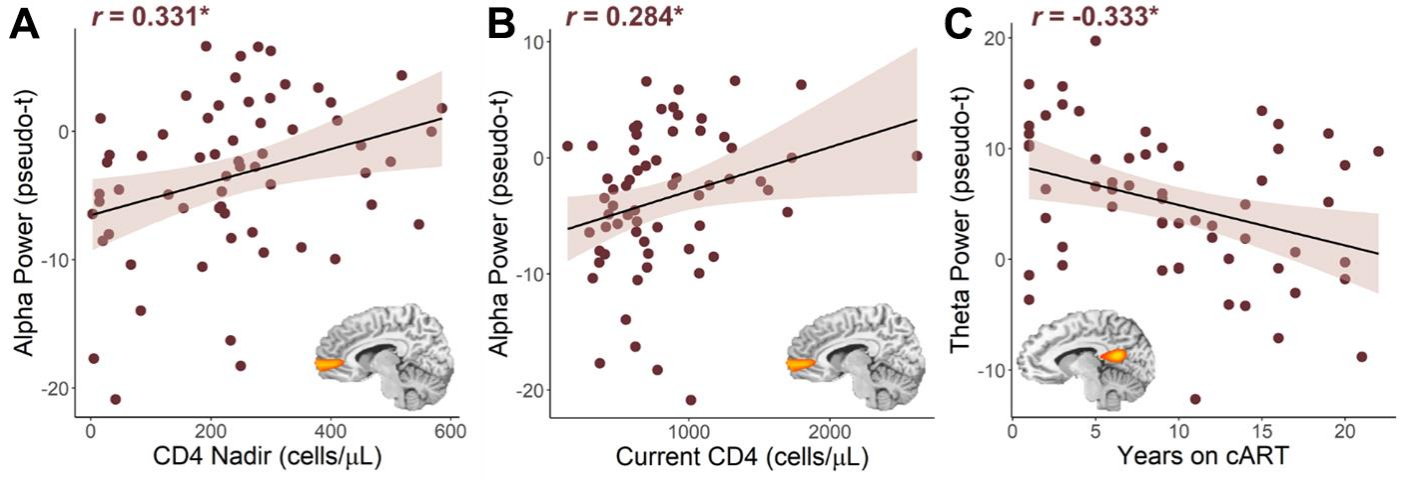
**Relationship between HIV metrics and oscillatory alpha and theta power.** Lower nadir CD4 counts (**A**) and lower current CD4 counts (**B**) were associated with stronger (i.e., more negative) oscillatory alpha power in the right medial prefrontal cortex. (**C**) The longer PWH had been on combination antiretroviral therapy (cART), the weaker their oscillatory theta power in the left posterior cingulate cortex. *p < 0.05.

Next, we wanted to probe how these distinct neural oscillatory responses were related to clinical indices of HIV (i.e., CD4 nadir, current CD4 counts, duration on cART, disease duration). Pseudo-*t* values were extracted from peak voxels identified in the whole-brain interaction analyses and subjected to additional testing. Regarding clinical HIV metrics, we found that lower CD4 nadir counts (cells/μL; *r* = 0.331, *p* = 0.009; Figure 4A) and lower current CD4 counts (*r* = 0.284, *p* = 0.025; Figure 4B) were associated with stronger (i.e., more negative) alpha power in the left mPFC. Conversely, we found that weaker theta power in the left PCC was associated with being on cART for a longer period of time (*r* = -0.333, *p* = 0.010; Figure 4C), but was not significantly associated with HIV disease duration (*r* = -0.229, *p* = 0.080).

## DISCUSSION

Previous work has identified aberrancies in neural dynamics during visuospatial processing in cognitively impaired PWH [11] and showed that such aberrations follow a distinct trajectory with chronological age [12]. However, to our knowledge, no studies to date have examined the relationship between such neural oscillatory dynamics and DNA methylation measures of biological aging in PWH relative to seronegative controls. Using sophisticated neuroimaging and statistical methods, we found that biological age and HIV status uniquely interact with the neural oscillatory dynamics serving visuospatial processing. In particular, we identified differential associations between biological age and theta and alpha neural oscillatory power among PWH and seronegative controls in the left PCC and right mPFC, respectively. Previous studies have reported evidence for an interaction between chronological age and HIV infection on neural and cognitive functioning [10, 12, 13, 16, 20]. Further, a systematic review highlighted that 45% of cross-sectional studies identified premature aging among PWH relative to age-matched seronegative controls, and 75% of longitudinal studies demonstrated that PWH exhibited accelerated aging [27]. However, none of these studies have investigated biological age as a factor. Thus, these findings are the first to identify the interaction between biological age and HIV status on neural oscillatory dynamics serving cognitive function, and more specifically, visuospatial processing. Thus, the present study adds a critical component to this literature and extends previous findings by relating previously reported abnormalities in neuronal oscillations to biological epigenetic aging. Notably, the two key regions identified in the present study (i.e., the PCC and mPFC) have been associated with diminished gray matter volume in PWH relative to seronegative controls [10, 28], and were previously linked to accelerated biological aging in a structural neuroimaging study [10].

The PCC is highly metabolically active and connected with many areas across the cortex, though it is typically less active during cognitively demanding tasks [29]. The PCC is thought to play a critical role in regulating the focus of attention both internally and externally [29], and at least one study showed that neural response strength in the PCC scales with cognitive load in healthy individuals [30]. Further, those who fail to adequately reduce activity in the PCC while undergoing cognitively-demanding tasks often exhibit cognitive inefficiencies [31–35], and studies have shown that healthy older individuals express stronger PCC activity while undergoing an externally directed task relative to younger individuals [36–38]. Further, HIV has been linked to reduced regional and global cortical myelin [39] and weaker activity in the PCC [40–44], which has been associated with poorer cognitive functioning in PWH [45]. In contrast to the PCC, the mPFC is critical for executive functions including attention, cognitive flexibility, working memory, and decision-making [46]. Independent of executive functions, there is a strong relationship between deterioration in information processing speed and age-related changes in the mPFC [47].

By integrating epigenetic data with neural oscillatory theta measures during a visuospatial processing task, we identified an HIV-by-biological age interaction, such that theta power in the left PCC was weaker with older biological age in PWH but was stronger with older biological age in seronegative controls. This may reflect a progressive deficit in recruiting greater neural resources to compensate for age-related decreases in neural efficiency in biologically older PWH. In line with the CRUNCH hypothesis [26], biologically older seronegative controls were able to sufficiently recruit more neural resources to compensate for biological age-related decreases in neural efficiency during this relatively simple visuospatial processing task, while biologically older PWH appeared unable to adequately recruit the extra neural resources needed to meet the cognitive demands of the task [13, 26]. Finally, in further support for the notion that weaker theta activity with older biological age is pathological, we found that those who had been on cART longer had weaker (i.e., less optimal) theta power in the left PCC, which may reflect legacy effects of earlier antiretrovirals or simply cumulative effects of cART exposure. In contrast to theta, PWH exhibited stronger (i.e., more negative) alpha oscillations in the mPFC with greater biological age, which we propose was likely compensatory given the overall pattern of relationships with HIV clinical indices. Specifically, the stronger oscillatory alpha responses in the mPFC were associated with lower nadir CD4 counts and lower current CD4 counts, which may reflect that those with the most severe legacy effects and/or weaker immune systems rely on the strongest compensatory activity to complete this relatively simple visuospatial processing task. In sum, we suggest that HIV-related changes to theta and alpha neural oscillations are closely associated with measures of biological age at a molecular level. Of note, epigenetic age of brain tissue and of peripheral blood measured via DNA methylation have been shown to correspond highly with one another [22, 48].

Aging in the brain has largely been studied using only chronological age, though biological age has been showed to be associated with greater mortality risk [21]. Therefore, our study not only illuminates the nature of HIV-related alterations to neural oscillations, but also suggests that epigenetic measures of biological age may be important in understanding HIV-related neural aberrations. Such increases in biological age in PWH may be related to legacy effects, cART exposure, viral reservoirs in the CNS and periphery, or other factors, any of which could lead to inflammation, cellular damage, and ultimately epigenetic modifications that reflect accelerated biological age, potentially driving changes in neural structure and function [10]. More specifically, epigenetic modifications to immune cells are thought to contribute most to accelerated biological aging and cognitive impairment in PWH by way of HIV-infected monocyte and macrophage lineages crossing the blood brain barrier, infecting microglia and astrocytes in the brain, thereby inducing a neuroinflammatory state that results in synaptic and neuronal loss and degeneration of brain circuits, thus contributing to HIV-related cognitive impairment and accelerated biological aging [49]. However, further investigation is warranted to establish the causality of this relationship. While these findings replicate previous epigenetic changes that occur with HIV and extend structural imaging findings of a link between HIV and biological aging in the brain to neural function, many questions remain for future studies to investigate.

Despite the novelty of this study, there were some limitations that govern the generalizability of the results. For example, we only recruited PWH who were virally suppressed, receiving an effective cART regimen, and had no diagnosed neurologic/psychiatric comorbidities, including substance use disorders. We also did not assess the impact of socioeconomic status, health behaviors, or comorbidities such as cardiovascular (e.g., hypertension) and metabolic (e.g., diabetes) diseases, which should be assessed in the future. These limitations may also be why the difference in biological age that we observed in PWH was smaller than that reported in previous studies [50].

In conclusion, our study identified differences in the relationship between biological age and theta and alpha oscillatory neural activity serving visuospatial processing in PWH and seronegative controls. Specifically, epigenetic alterations revealed that biologically older PWH were not able to adequately recruit neuronal populations underlying oscillatory theta responses in the left PCC. Conversely, PWH exhibited stronger oscillatory alpha power in the right mPFC, and this activity appeared to be compensatory and may underlie their ability to adequately perform the visuospatial processing task. In sum, these findings provide compelling evidence linking epigenetic biological aging and aberrations in neural oscillatory activity in PWH, suggesting that biological aging may underlie some of the key neurological findings in the neuroHIV literature.

## MATERIALS AND METHODS

### Participants

We studied participants between the ages of 22 to 72 years who were originally enrolled as part of a larger project examining chronological aging in HIV (R01-MH103220; see Table 1). PWH were required to be on an effective cART regimen and have an HIV RNA viral load of less than 50 copies/mL within three months of participation in the study. All controls were confirmed seronegative using the OraQuick *ADVANCE*® Rapid HIV Antibody Test at the time of neuropsychological testing. Exclusion criteria for the study included any diagnosed neurological or psychiatric disorder (except HIV-associated cognitive impairment), any medical illness associated with CNS dysfunction (other than HIV), history of head trauma, current substance use disorder, the presence of metallic implants that could affect MEG signal quality or MRI safety, and pregnancy. Additionally, we excluded participants who did not have DNA methylation data, had artifactual or missing MEG data, or had incidental findings (e.g., tumor). All participants completed a neuropsychological assessment in accordance with the Frascati criteria [51] that assessed for HIV-associated cognitive impairment. Raw scores were converted to demographically corrected scores using published normative data [52–56] and the resulting T-scores of similar assessments were averaged together to create domain composite scores [28]. These are reported in Table 1.

### DNA methylation and biological age

Peripheral whole blood samples were collected from each participant using BD Vacutainer EDTA tubes to assess DNA methylation metrics of predicted biological age based on the Hannum, Horvath, and consensus models, as previously described by Lew and colleagues [10] and Spooner and colleagues [57, 58]. Briefly, DNA was purified from whole blood using DNeasy blood tissue extraction kits (Qiagen, Germantown, MD). Methylation analysis was performed using Infinium HumanMethylation450 BeadChip Kits (Illumina, San Diego, CA). Following hybridization, BeadChips were scanned using the Illumina HiScan System. All data were processed through the Minfi R processing pipeline [59]. Methylome data were downloaded from Hannum [23] and EPIC [60] (GEO: GSE40279 and GSE51032), and these data were processed together along with methylation data generated from the larger study mentioned above. Beta values were extracted and quantile normalized using Minfi; cell counts were estimated using the estimateCellComposition function and resulting normalized beta values were adjusted for cell types [50, 61]. All data was then normalized using a modified BMIQ procedure provided by Horvath [22]. The gold standard was set to the median beta observed in the Hannum study [23]. For the current study, the “consensus model” of predicted biological age (i.e., both Hannum and Horvath predictions) was used, as this has been shown to outperform either prediction model in isolation [50]. In addition, relative age acceleration was calculated using the residuals of a regression of the consensus model of predicted biological age on chronological age for our sample.

### MEG experimental paradigm

Participants underwent a 12-minute visuospatial discrimination task (Figure 1), which has been validated and described in previous work [11, 12, 62, 63]. Participants were seated in a magnetically-shielded room and were instructed to fixate on a centrally located crosshair with a variable ISI (range: 1900 – 2100 ms) followed by the presentation of an 8 × 8 grid for 800 ms in one of four positions laterally offset by 75% relative to the crosshair: top left, top right, bottom left, or bottom right. Participants were instructed to respond with their index finger via button response if the grid was positioned to the left and with their middle finger if the grid was positioned to the right. The trials were equally divided between the four different positions and pseudorandomized. Reaction time and accuracy measures were collected and used for behavioral analysis. Independent samples *t*-tests were used to assess differences in reaction time and accuracy between seronegative controls and PWH, and a Pearson correlation was used to examine the association between reaction time and biological age.

### MEG and MRI data acquisition

Functional MEG data were collected using an Elekta/MEGIN MEG system (Helsinki, Finland) equipped with 306 sensors (204 planar gradiometers, 102 magnetometers) using a 1 kHz sampling rate and an acquisition bandwidth of 0.1-330 Hz in a one-layer magnetically shielded room with active shielding engaged. Prior to MEG acquisition, four coils were attached to the participant’s head and localized along with fiducial and scalp surface points using a three-dimensional (3D) digitizer (FASTRAK 3SF0002, Polhemus Navigator Sciences, Colchester, Vermont). Once the participants were positioned for MEG recording, an electric current with a unique frequency label (e.g., 322 Hz) was fed to each of the four coils, thus inducing a measurable magnetic field and thereby allowing each coil to be localized in reference to the MEG sensor array throughout the recording session. Structural T1-weighted images were collected using a 3D-fast-field echo sequence on a Philips Achieva 3.0T X-Series scanner with an eight-channel head coil. The parameters for the 3D-fast-field echo sequence were as follows: TR: 8.09 ms; TE: 3.7 ms; field of view: 24 cm; matrix: 256 × 256; slice thickness: 1 mm with no gap; in-plane resolution: 0.9375 × 0.9375 mm; sense factor: 1.5.

### MEG and MRI processing

MEG and MRI data processing closely followed previously reported pipelines [11, 12, 64, 65]. The structural MRI data were aligned parallel to the anterior and posterior commissures and transformed into standardized space. MEG data were subjected to environmental noise reduction and corrected for head motion using the signal space separation method with a temporal extension [66]. Only data from the 204 planar gradiometers were used for further analysis. All MEG and MRI data were further processed in BESA (Research: Version 7.0; MRI: Version 2.0; Statistics: Version 2.0). Cardiac and ocular artifacts were regressed out of the MEG data using signal space projection (SSP) [67].

### MEG time-frequency transformation

The continuous magnetic time series was then filtered between 0.5 – 200 Hz with a 60 Hz notch filter. Epochs were 2700 ms, with the baseline extending from -400 to 0 ms prior to visual stimulus onset. Only trials with correct responses were considered for further analysis. Epochs containing artifacts were rejected using a fixed threshold method that was set per participant and supplemented with visual inspection. Briefly, in MEG, the raw signal amplitude is strongly affected by the distance between the brain and the MEG sensor array, as the magnetic field strength falls off sharply as the distance from the current source (i.e., brain) increases. To account for this source of variance across participants, as well as other sources of variance, we used an individualized threshold based on the signal distribution for both amplitude and gradient to reject artifacts. The average amplitude threshold across all participants was 1021.16 (SD = 280.63) fT/cm, the average gradient threshold was 208.92 (SD = 102.17) fT/(cm*ms), and an average of 203.66 (SD = 20.30) trials out of the original 240 were used for further analysis. The number of trials included in the final MEG analyses did not statistically differ by group (*t* = 0.53, *p* = 0.60).

### Sensor-level statistics

We then transformed the artifact-free epochs into the time-frequency domain (resolution: 2 Hz, 25 ms) using complex demodulation [68, 69]. Each sensor’s spectral power estimations were averaged over trials to produce time-frequency plots of mean spectral density, which were then normalized by the baseline power of each respective bin, calculated as the mean power from -400 to 0 ms. The time-frequency windows for subsequent source imaging were identified using a stringent two-stage statistical approach that utilized paired-sample *t*-tests on each pixel in the spectrogram (per sensor) at the first stage, followed up with cluster-based nonparametric permutation testing at the second level. This testing was conducted across all participants and the entire frequency range (4 – 100 Hz) and used an initial cluster threshold of *p* < 0.001 and 10,000 permutations. These methods are described in depth in our recent publications [11–13, 65]. The resulting time-frequency clusters that survived permutation testing were selected for source imaging analyses.

### MEG source imaging

Time-frequency resolved source images were computed using the dynamic imaging of coherent sources (DICS) beamformer to image oscillatory activity in the time-frequency windows of interest per participant [70–72]. Following convention, we used task and baseline periods of equal duration and bandwidth for each time-frequency cluster identified in the sensor analysis to derive noise-normalized source power per voxel for each participant. The resulting pseudo-t maps represent noise-normalized source power differences (i.e., active versus baseline) per participant and voxel (resolution: 4 × 4 × 4 mm). These maps were then transformed into standardized space and spatially resampled by applying the same transform that was applied to the native space structural images per participant. Individual participant-level maps containing significant artifacts were excluded from further analysis.

### Whole-brain statistics

To probe the interaction between biological age and HIV status on neural oscillatory networks serving visuospatial processing, the spectrally specific whole-brain maps for each participant were used. Briefly, we first computed voxel-wise correlations between biological age and spectrally specific neural activity within each group (i.e., PWH vs. control) and then compared the resulting statistical maps by group using a whole-brain Fisher *Z* transformation [12, 73]. To account for multiple comparisons, a significance threshold of *p* < 0.005 was used for the identification of significant clusters in all whole-brain statistical maps, accompanied by a cluster (*k*) threshold of at least 45 contiguous voxels (i.e., 2,880 mm^3^ of brain tissue). All whole-brain statistical analyses were computed using a custom function in MATLAB (MathWorks; Natick, Massachusetts) and other statistical analyses were conducted in IBM SPSS v.25.

### Data availability

The data that support the findings of this study are available upon reasonable request from the corresponding author, Dr. Tony W. Wilson.
